# A ~40-kb flavi-like virus does not encode a known error-correcting mechanism

**DOI:** 10.1073/pnas.2403805121

**Published:** 2024-07-17

**Authors:** Mary E. Petrone, Joe Grove, Julien Mélade, Jonathon C. O. Mifsud, Rhys H. Parry, Ezequiel M. Marzinelli, Edward C. Holmes

**Affiliations:** ^a^Sydney Institute for Infectious Diseases, School of Medical Sciences, The University of Sydney, Sydney, NSW 2006, Australia; ^b^Laboratory of Data Discovery for Health Limited, Hong Kong Special Administrative Region, China; ^c^MRC-University of Glasgow Centre for Virus Research, Glasgow G61 1QH, United Kingdom; ^d^School of Chemistry and Molecular Biosciences, The University of Queensland, Brisbane, QLD 4067, Australia; ^e^School of Life and Environmental Sciences, The University of Sydney, Sydney, NSW 2006, Australia

**Keywords:** evolution, virology, metatranscriptomics, error threshold, Flaviviridae

## Abstract

It is commonly held that there is a fundamental relationship between genome size and error rate, manifest as a notional “error threshold” that sets an upper limit on genome sizes. The genome sizes of RNA viruses, which have intrinsically high mutation rates due to a lack of mechanisms for error correction, must therefore be small to avoid accumulating an excessive number of deleterious mutations that will ultimately lead to population extinction. The proposed exceptions to this evolutionary rule are RNA viruses from the order *Nidovirales* (such as coronaviruses) that encode error-correcting exonucleases, enabling them to reach genome lengths greater than 40 kb. The recent discovery of large-genome flavi-like viruses (*Flaviviridae*), which comprise genomes up to 27 kb in length yet seemingly do not encode exonuclease domains, has led to the proposal that a proofreading mechanism is required to facilitate the expansion of nonsegmented RNA virus genomes above 30 kb. Herein, we describe a ~40 kb flavi-like virus identified in a *Haliclona* sponge metatranscriptome that does not encode a known exonuclease. Structural analysis revealed that this virus may have instead captured cellular domains associated with nucleic acid metabolism that have not been previously found in RNA viruses. Phylogenetic inference placed this virus as a divergent pesti-like lineage, such that we have provisionally termed it “Maximus pesti-like virus.” This virus represents an instance of a flavi-like virus achieving a genome size comparable to that of the *Nidovirales* and demonstrates that RNA viruses have evolved multiple solutions to overcome the error threshold.

## Significance

A hallmark of RNA viruses is their small genome size, a feature attributed to the inability of their polymerase to correct errors. Because the accumulation of mutations results in the decline of fitness, RNA viruses must maintain small genomes to avoid extinction. Members of the *Nidovirales* are the sole exception because some have stolen error-correcting domains from cellular hosts. Here, we present an instance of a nonsegmented RNA virus outside of the *Nidovirales* that supports a genome size above 30 kb. This virus encodes a ~40 kb genome and falls within the *Amarillovirales*. Notably, we did not detect a known proofreading mechanism in its genome, suggesting that multiple evolutionary strategies are available for overcoming the error threshold.

RNA viruses have traditionally been characterized by small, compact genomes, often close to 10 kb in length (1). The longest RNA virus genome documented to date is ~47 kb (2), considerably shorter than those seen in DNA viruses whose genomes can exceed 2.5 Mb (3). Despite the explosion in RNA virus discovery following the advent of metagenomic sequencing (4–7), the maximum genome sizes of most families of RNA viruses have remained relatively stable, with the longest nonsegmented genomes (i.e., >30 kb) consistently falling within families from the order *Nidovirales*. Some segmented viruses have been found to achieve comparable lengths, such as Dendrolimus punctatus cypovirus, which encodes a genome of ~33 kb across 16 segments ranging from 700 bp to 4,100 bp in length (8).

The most popular theory for the restricted genome sizes of RNA viruses is that it stems from the mutational burden associated with replication via an error-prone RNA polymerase [i.e., RNA-dependent RNA polymerase (RdRp) or reverse transcriptase] (9). Because these enzymes usually lack any form of error correction such as proofreading, longer genomes are expected to accumulate more mutations per genome replication. As most mutations are deleterious (or even lethal) (10), a high error rate will greatly reduce virus fitness, eventually leading to population extinction. Consequently, an upper cap on genome sizes reflects an “error threshold,” with high mutation rates necessarily resulting in smaller genomes.

The existence of a mutation-driven cap on virus genome size is supported by two lines of evidence. First, the treatment of experimental populations of RNA viruses with mutagenic agents, such as 5-azacytidine and 5-fluorouracil, results in major fitness losses as expected if these viruses reside close to the error threshold (11). Indeed, fitness losses resulting from the use of antiviral mutagenic agents such as ribavirin and molnupiravir (12) underpin viral therapies based on the induction of so-called “lethal mutagenesis” (13, 14). Second, members of the *Nidovirales,* which include RNA viruses with the largest known genomes (2), have acquired a unique proofreading mechanism. The *Coronaviridae* encode endo- and exoribonucleases (such as nsp14-ExoN) that are essential for replication (15) and that correct replication errors (16, 17). Similarly, members of the *Roniviridae* utilize NAD and ADP-ribose (NADAR) domains which are associated with RNA repair (18). Reducing the error rate per nucleotide should, in theory, have enabled nidovirus genomes to expand in size. Indeed, the discovery of Nam Dinh virus, a ~20 kb nidovirus that is distantly related to the *Coronaviridae* and the *Roniviridae* and that encodes an exoribonuclease, further supports this hypothesis (19).

Although attractive, the error threshold theory for virus genome size was challenged by the discovery of the so-called “large-genome flavi-like” viruses (LGFs) that possess genomes up to 27 kb in length (20, 21) and hence comprise the second-longest group of nonsegmented RNA virus genomes documented to date. Importantly, however, the LGFs do not encode an exoribonuclease or similar domain, but rather a stabilizing methyltransferase domain. This domain has been proposed as a mechanism for promoting genome size expansion but is not directly involved in proofreading (22). Previously, no nonsegmented virus outside of the order *Nidovirales* had been identified with a genome exceeding 27 kb, and it was not known whether longer genomes could be supported in the absence of a known proofreading mechanism.

Herein, we describe a divergent, flavi-like virus with a genome of ~40 kb that does not encode an exoribonuclease or recognizable proofreading domain. This finding provides insights into the evolution of the *Flaviviridae* and demonstrates that exonuclease domains are not required to support RNA virus genomes above 27 kb.

## Results

### Identification of a Large and Highly Divergent Marine Flavi-Like Virus.

We identified a ~40 kb flavi-like virus in a sea sponge (order Haplosclerida) metatranscriptome. This sponge, along with 72 invertebrate specimens, was collected from Chowder Bay, Sydney Harbour, Sydney, Australia, on October 20, 2022. Assessment of the true host association of this virus was challenging due to the complexity of the sequencing library (*SI Appendix*, Fig. S1*A*), which in turn reflected the heterogeneous composition of the ecosystem from which it was collected (*SI Appendix*, Fig. S1*B*). Despite this, over 74% of rRNA reads in this library shared high sequence similarity with rRNA of sponge, particularly those belonging to the genus *Haliclona,* order Haplosclerida (Dataset S1). Given that the morphology of the specimen was consistent with *Haliclona* (*SI Appendix*, Fig. S1 *B*, *Inset*) and this genus has been previously documented in Sydney Harbour (ozcam.ala.org.au), we concluded that a *Haliclona* sponge was the most probable host organism of this virus.

**Fig. 1.**
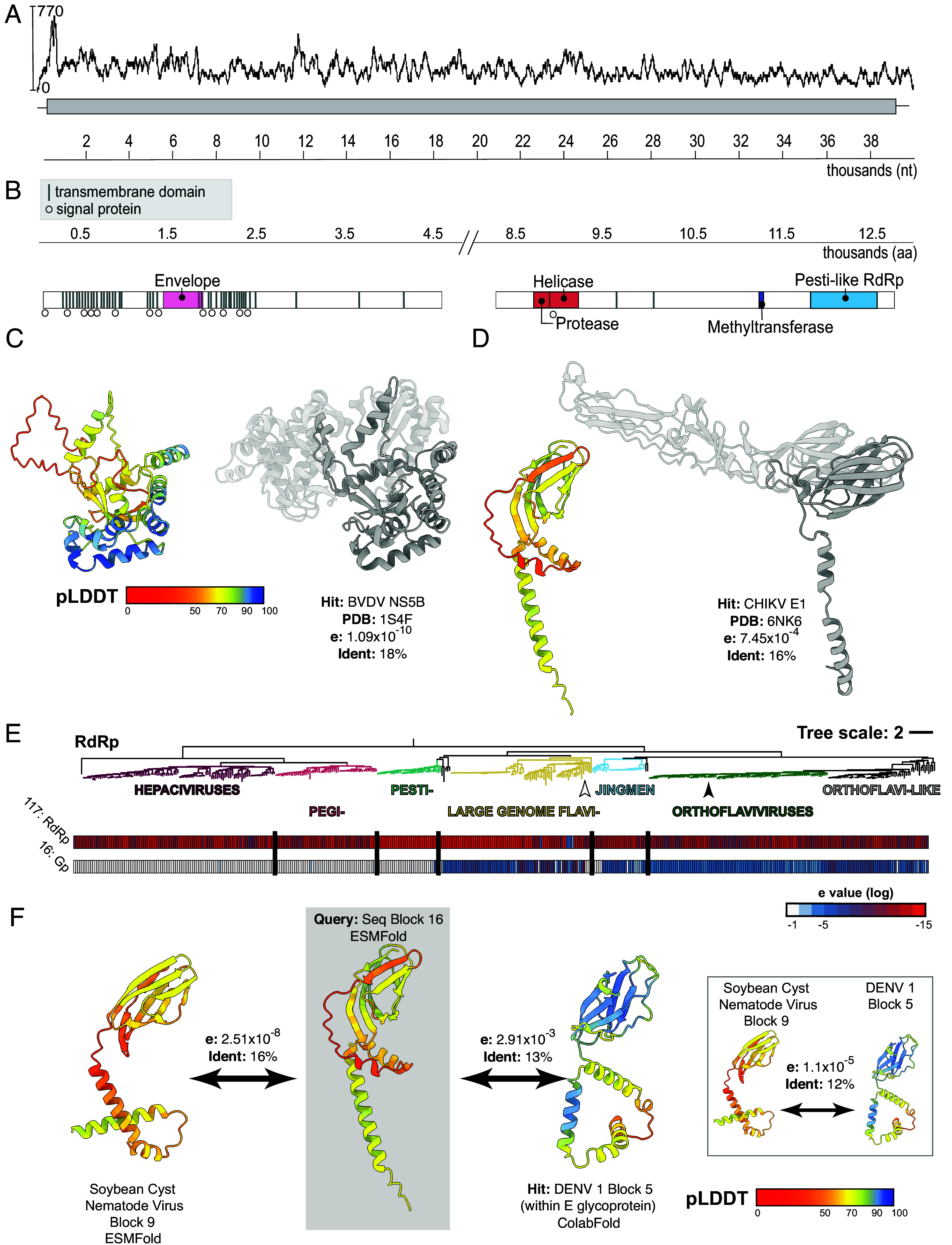
Characterization of canonical *Flaviviridae* features in a highly divergent, large-genome flavi-like virus. (*A*) Sequencing coverage of nucleotide sequence. The *y*-axis measures sequencing depth. Reads were mapped using BBMap (26) and the coverage plot was generated in Geneious Prime (www.geneious.com). (*B*) Functional domains identified using sequence-based and protein fold-based homology. (*C*) Structural prediction of the putative NS5 protein. *Left*: ESMFold structure prediction for polyprotein sequence block 117 (amino acid residues 11,700 to 12,000), color-coded by prediction confidence (predicted Local Distance Difference Test, pLDDT) as shown in the key. *Right*: homologous BVDV NS5B structure (PDB:1S4F) returned by Foldseek as the top hit. Expected value (e-value) and percentage sequence identity are shown. Regions of structure not aligned by Foldseek are transparent. (*D*) Homology between sequence block 16 (with putative glycoprotein region) and Chikungunya virus (CHIKV) E1 structure (PDB: 6NK6), the top hit returned by Foldseek, displayed as in (*C*). (*E*) RdRp maximum likelihood phylogeny for the *Flaviviridae* as described by Mifsud et al. (27). The tree is labeled and colored to indicate major viral genera and groups. The heatmap displays log transformed e-values (color coded as shown in the key) demonstrating the distribution of homologues to block 117 (RdRP) and block 16 (glycoprotein) within the *Flaviviridae* protein foldome. (*F*) Example structural homologues to block 16 from a large-genome flavivirus (Soybean cyst nematode virus) and a canonical orthoflavivirus (Dengue virus 1 [DENV 1]); e-values and sequence identities are provided. The inset demonstrates structural similarity between these two hits. The location of the Soybean cyst nematode virus and DENV 1 are indicated on the *Flaviviridae* phylogeny (*E*) with white and black arrowheads, respectively.

This virus exhibited clear, yet minimal, sequence similarity with members of the *Flaviviridae*. It was most closely related to Fushun flavivirus 1 (length = 15 kb), with which it shared 25.7% sequence similarity (e-value = 1.31e-23, Reference Viral Database). Like other *Flaviviridae*, it comprised a single polyprotein (38 kb) and long 5′ and 3′ untranslated regions (UTRs) (488 nt and 1,277 nt, respectively) (Fig. 1*A*). The polyprotein sequence was complete, containing no premature stop codons and exhibited a strong AT bias (64%). Its abundance in the sequencing library was low, comprising less than 0.01% of non-rRNA reads (56,371 reads mapped to the flavi-like virus contig), but its sequencing coverage was robust, with a mean depth of 139 (SD = 13.2) (Fig. 1*A*). We recovered identical contigs with three independent assembly methods [MEGAHIT v1.2.9 (23), SPAdes v3.15.5 (24), and Trinity v2.8.6 (25)].

Fragments of this virus were detected in two of the three other libraries of samples collected from the same piling (Bryozoa, <0.0001% non-rRNA reads; sponge, 0.015% non-rRNA reads) [*SI Appendix*, Fig. S2, (28)]. To our knowledge, this is the longest known flavi-like virus, nearly 1.5 times the length of previously documented LGFs. It is also the longest known RNA virus outside of the order *Nidovirales* and the only known RNA virus over 30 kb to encode a single polyprotein.

To assess whether this putative virus was replicating in our environmental sample, we performed strand-specific PCR. Because the replication of RNA viruses involves the synthesis of negative-strand intermediates, both positive- and negative-sense strands would be produced during viral replication. Both strands could be amplified using RT-PCR (*SI Appendix*, Fig. S3) and quantified with qPCR (positive strand: 2.28 × 10^6^ copies/mL, negative strand: 1.10 × 10^3^ copies/mL).

Despite its length and low sequence similarity to the *Flaviviridae*, this virus possessed characteristics of canonical flavivirids. To explore genome organization and annotate protein function we used both sequence-based approaches (BLASTp, InterProScan, SuperFamily, NCBI CD-Search, and NCBIfam) and protein structure prediction followed by fold-based homology searches. For the latter, the polyprotein sequence was broken into overlapping 300 residue blocks for structure prediction, using ColabFold-AlphaFold2 (29, 30) and ESMFold (31), and queried using the Foldseek (32) server. The putative NS2/3 protein featured a consecutive serine protease and helicase pair (Fig. 1*B*). Manual annotation showed that the serine protease included the typical Ser (nucleophile), His (base), Asp (acid residue) catalytic triad (*SI Appendix*, Fig. S4*A*). Similarly, the putative NS5 RdRp protein was pesti-like in both sequence and predicted structure (Fig. 1*C*) and retained the highly conserved GDD motif (*SI Appendix*, Fig. S4*B*). Like other LGFs, a methyltransferase domain was present within the putative NS5. However, we found no regions that were similar to nido-like endo/exonucleases or NADAR domains (Fig. 1*B*).

An envelope protein surrounded by signal peptides and probable N-linked glycosylation sites was detected near the 5′ terminal (Fig. 1 *B* and *D* and *SI Appendix*, Fig. S5). ESMFold protein structure prediction yielded a domain of intermediate prediction confidence toward the C terminus of this region. This domain exhibited similarity to domain III of a class II fusion protein, with strongest homology to glycoproteins from bunya-(Gc) and alphaviruses (E1) rather than the E glycoprotein found in orthoflaviviruses (Phyre2: Rift Valley Fever virus, HHPred: Thrombocytopenia syndrome virus, Foldseek: Chikungunya virus, Dataset S2). This is consistent with the highly divergent nature of this protein, as this region exhibited only 16% identity with its closest homolog (Fig. 1*D*).

However, viral proteins are underrepresented in fold-based homology search servers such as Foldseek. To address this, we previously generated a protein foldome for the *Flaviviridae* composed of predicted structures for >450 viral species, all processed using the 300-residue sequence block strategy described above (27). We queried this database for similarity to the RdRp and glycoprotein folds identified in the sponge flavi-like virus using Foldseek (Fig. 1 *E* and *F*). Being highly conserved, RdRp homologues were found in all species, with highest similarity residing in the pestiviruses and LGFs. Glycoprotein homologues were present across the large-genome flavi-, jingmen- and orthoflaviviruses, where similarity corresponded to E glycoproteins. Glycoprotein homologues could not be found in the pesti-, pegi- or hepaciviruses, which possess the E1E2 glycoproteins that are structurally distinct from the E glycoprotein (33). We concluded that this virus expresses an E-like glycoprotein homologous to other LGFs. Together, the quality of the sequence and the organization of the putative genome suggested that this unusually large virus was exogenous and related, albeit distantly, to the *Flaviviridae*.

### Divergent, Cellular-Associated Domains in an RNA Virus Genome.

**Fig. 2.**
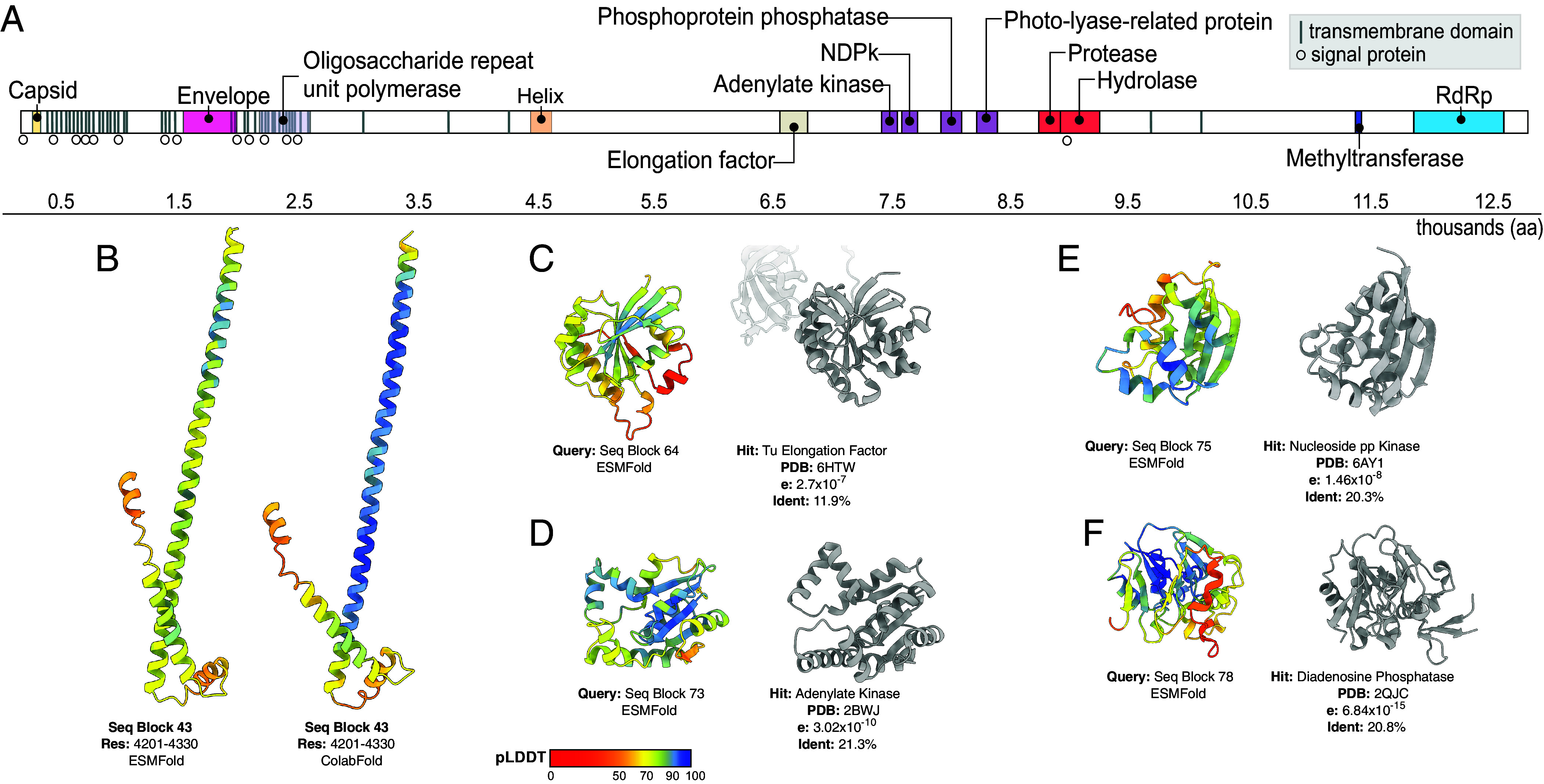
Large-genome flavi-like virus polyprotein encodes a complement of noncanonical functional domains. (*A*) Annotation of domains detected using a combination of sequence and structure-based analyses: InterProScan, DeepTMHMM (transmembrane domains), SignalP (signal peptidases), Pfam, CDD, and Foldseek. Domain annotations reflect domains sharing the closest structural homology to the putative viral domains. The polyprotein is scaled to thousand amino acids. NDPk: nucleoside diphosphate kinase. Helical domain: helical domain of unknown function. (*B*) Inferred ESMFold and ColabFold structures of a helical domain of unknown function [found in sequence block, (34)], color-coded by prediction confidence (pLDDT) as shown in the key (*C*–*F*) ESMFold structures of the stated sequence blocks alongside their cognate, top representative Foldseek hits, expected value, and percentage sequence identity are provided. Regions of structure not aligned by Foldseek are transparent.

Given its size, we hypothesized that this virus would encode noncanonical flavivirus domains, particularly those that would help support the replication of such a large RNA genome. Accordingly, using both sequence- and structure-based approaches, we found three notable features: a divergent extended helical domain (amino acid pos. 4,346 to 4,475), a Tu elongation factor (pos. 6,419 to 6,586), and a nucleic acid metabolism cassette (pos. 7,286 to 8,206) (Fig. 2*A*).

The potential functions of these domains were uncertain. The role of the extended helical domain could not be determined. Both ESMFold and ColabFold structures were of intermediate to high confidence and were in excellent agreement, supporting their veracity. However, this feature did not share detectable homology with known proteins in both ESMFold and ColabFold predictions (Fig. 2*B*). Its position in the genome proximally downstream of the envelope protein could be consistent with host response antagonism like the canonical NS1 (35), although experimental analysis is required to test this. In cellular organisms, Tu elongation factors (Fig. 2*C*) principally facilitate translation (36). This may be particularly beneficial to a virus with a very long polyprotein, but Tu elongation factors are also thought to exhibit “moonlighting” (i.e., noncanonical) functions (36). In this instance the protein was highly divergent, exhibiting no detectable sequence similarity to any other protein in the NCBI nr database, including from cellular organisms, which may reflect functional divergence. We identified related domains in the genomes of some *Megaviricetes* and *Caudoviricetes* (i.e., DNA bacteriophage) when we screened the Reference Proteomes database with HMMER (37), although we found no homologues in RNA viruses. The predicted structure shared homology with eukaryotic and bacterial elongation factors, but it was most closely related (11.9% identity) to an ancestrally reconstructed bacterial Tu Elongation Factor (Fig. 2*C*). However, it should be noted that lower-confidence/low-frequency Foldseek hits suggest other potential identities for this fold (e.g., uridine phosphorylase; *SI Appendix*, Fig. S6).

Elements of the nucleic acid metabolism cassette were more conserved. This region comprised an adenylate kinase, a nucleoside diphosphate kinase (NDPk), a phosphoprotein phosphatase, and a photolyase-related protein upstream of the NS2/3 protease-helicase pair (Fig. 2 *A* and *D*–*F*). Among these domains, NDPks are the most highly conserved across all kingdoms of life (38) and have been found in some giant DNA viruses and bacteriophage (39). They are involved in RNA synthesis but have not previously been documented in RNA viruses. Interestingly, the flavi-associated NDPk domain was more closely related to those in cellular organisms than to those in other viruses, sharing up to 30% sequence similarity to homologous domains in bacteria and eukaryotes. To search for related domains in viruses, we screened the NCBI nonredundant (nr) protein database restricted to viruses (taxid: 10239) using the flavi-associated NDPk as input. This returned no results, indicating that the flavi-associated NDPk domain does not share detectable sequence similarity with its counterparts in DNA viruses and thus is not closely related. It also suggested that NDPk acquisition is a rare event in RNA virus evolution. Querying Reference Proteomes, UniProtKB, Swiss-Prot, PDB, and ColabFold databases using hmmsearch with the NDPk alignment as input also failed to detect this domain in any publicly available RNA virus genomes.

Phylogenetic analysis of the NDPk domain could not confidently resolve its evolutionary relationship to cellular organisms. Although it did not share detectable sequence similarity to homologous domains in DNA viruses, the flavi-like virus-associated domain shared <30% similarity to domains in Bacteria, Viridiplantae, Fungi, Archaea, Metazoa, and Hapista. Using the top 500 Blastp matches filtered for redundancy at a threshold of 90%, we inferred four phylogenetic trees from a combination of aligning and trimming methods (*SI Appendix*, Fig. S7). In three of the four phylogenies, the flavi-like virus-associated NDPk domain was most closely related to bacteria-associated domains, albeit with low support (*SI Appendix*, Fig. S7 *B*–*D*). Regardless of its origins, this domain constitutes the only known example of such a feature in the characterized RNA virosphere.

### Phylogenetic Support for Classification as a Large-Genome Flavi-Like Virus.

**Fig. 3.**
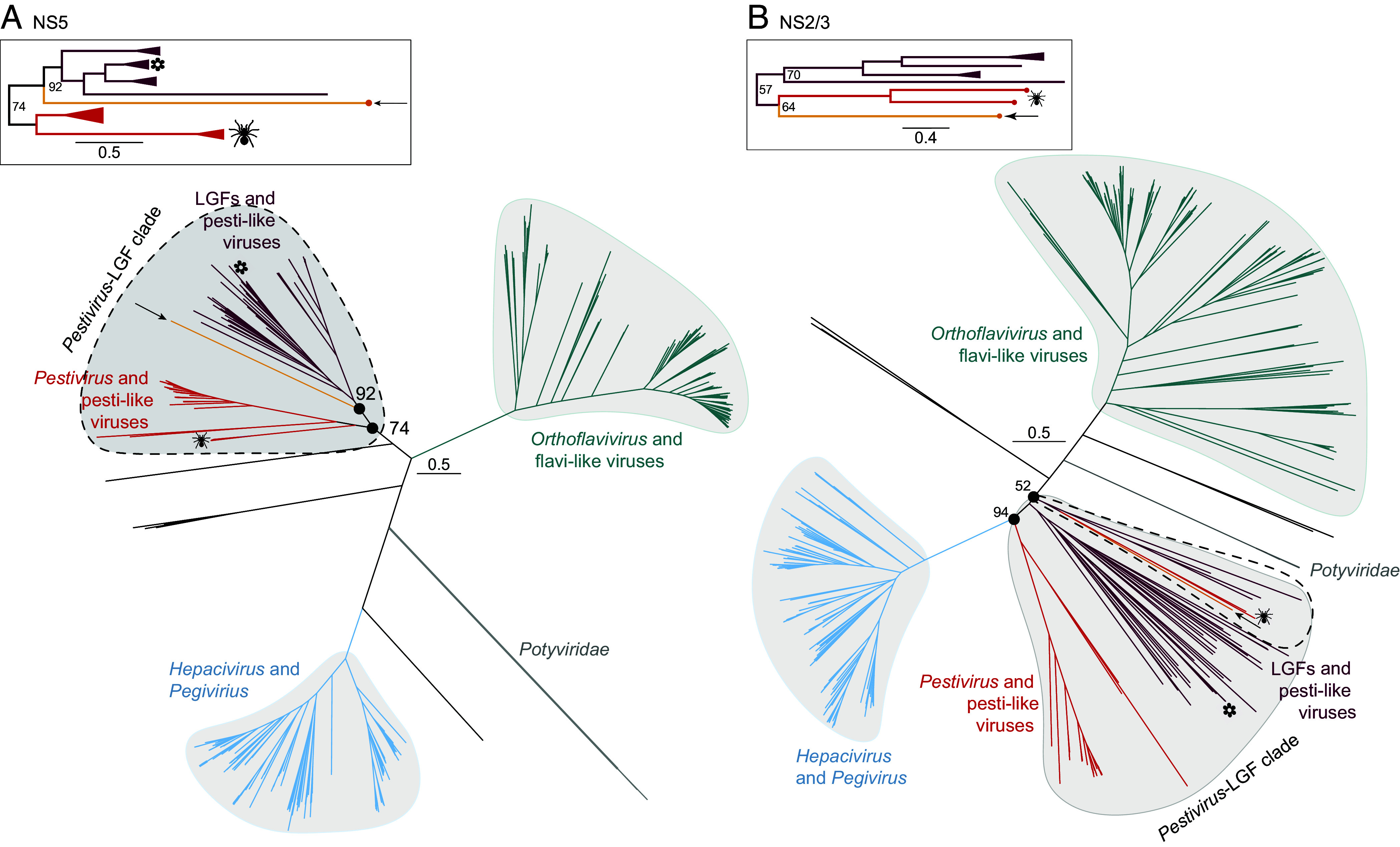
Phylogenetic analysis of the divergent flavi-like virus places it among the pesti-like viruses. Unrooted phylogenetic trees of *Flaviviridae.* (*A*) NS5 and (*B*) NS2/3. Arrows indicate the divergent flavi-like virus identified here. Flower icon indicates plant-associated flavi-like viruses. Spider icon indicates spider-associated pesti-like viruses. Ultrafast bootstrap values are shown as numbers at select nodes. *Insets* show the groups containing the divergent flavi-like virus and correspond to the regions encircled by dotted lines in the main figure. The topology of the insets reflects midpoint rooting of each tree in the main figures. Tree branches are scaled according to amino acid substitutions.

The unique and divergent protein complement encoded by this virus suggested that it could provide insights into the evolutionary history of the *Flaviviridae*. A family-level phylogenetic analysis placed both the putative NS2/3 and NS5 proteins with pesti-like viruses in a deep phylogenetic position (Fig. 3 *A* and *B*). We herein refer to this section of the tree as the “*Pestivirus*-LGF clade” that comprises the genus *Pestivirus*, pesti-like viruses, and LGFs. The placement of the divergent NS5 sequence at the base of the LGF clade was well supported (ufboot = 92), and the size of the viral genome was consistent with other members of this group (Fig. 3 *A*, *Inset*). In contrast, the NS2/3 proteins did not form a monophyletic group, although this topology did not have robust support (Fig. 3*B*). Here, the putative NS2/3 sequence from the divergent virus formed a sister group to two spider-associated pesti-like viruses (Xinzhou spider virus 3 and Shayang spider virus 4) (Fig. 3*B*, ufboot = 64). These spider-associated viruses were previously reported to be the most divergent members of the *Pestivirus* and pesti-like virus clade (40), and this relationship was reflected in our NS5 tree (Fig. 3*A*).

In an attempt to improve the accuracy of the phylogenetic position for both proteins, we realigned all NS2/3 and NS5 sequences belonging to the orthoflavivirus, pestivirus, pesti-like viruses, and LGFs using six combinations of sequence alignment and alignment trimming methods (Table 2). This did not yield more stable results for the placement of either region in the divergent virus (*SI Appendix*, Figs. S8 and S9). However, both amino acid sequences from the virus newly identified here consistently fell at the base of the *Pestivirus*-LGF clade, suggesting that this virus represents part of the deep evolutionary history of this group. Both midpoint rooting and rooting on the *Orthoflavivirus* clade consistently placed our virus within the sister clade to containing the genus *Pestivirus*. Importantly, it shared a closer phylogenetic relationship to animal-associated viruses than to plant-associated viruses, which form a monophyletic group. The placement of the divergent NS2/3 sequence, which formed a sister group to the spider-associated pesti-like viruses, is consistent with a history of virus–host codivergence. However, the broad cross-kingdom range of hosts associated with the *Pestivirus*-LGF clade indicates that the evolutionary history of this clade with respect to virus–host interactions is complex.

Together, our analyses support the conclusion that this virus is a large-genome sponge-infecting pesti-like virus, which we have provisionally named “Maximus pesti-like virus.”

## Discussion

We describe the largest known flavi-like virus and largest known RNA virus outside of the order *Nidovirales*. With a genome size of 39.8 kb, this virus surpassed the longest documented flavi-like virus to date by nearly 13 kb (20). Despite its large size, this virus possessed features that are characteristic of members of the *Flaviviridae*: an E-like glycoprotein, a protease/helicase pair, and a pesti-like RdRp. Although the E-like glycoprotein shared highest similarity to alpha- and bunyavirus glycoproteins, this is consistent with the notion that the class II membrane fusion machinery of the *Amarillovirales* (that contain the *Flaviviridae*), *Bunyavirales*, and *Martellivirales* evolved from a common ancestor (41). The polyprotein encoded a methyltransferase upstream of the RdRp, which is common throughout the *Flaviviridae* (22). The organization of the genetic components was also congruent with that of canonical *Flaviviridae*, a characteristic that differs from large nidoviruses, as these exhibit a flexible modularity in their genetic organization (2). Phylogenetic analysis placed the two most conserved proteins (NS2/3 and NS5) of this divergent virus deep within the *Pestivirus*-LGF clade, with a relatively close relationship to previously characterized LGFs. Although small portions of the genome shared sequence and structural homology to cellular proteins, we concluded that the virus was unlikely to be an endogenous viral element because the virus-associated sequences were highly divergent from their cellular homologues. Successful amplification and quantification of both positive- and negative-sense strands with PCR further supported this conclusion.

The identification of such a large flavi-like virus raises important questions. Does it possess a mechanism for correcting errors during replication? Or does it sustain a mutation rate similar to those seen in other flavivirids, and if so, how? We did not find evidence for nido-like endo- or exonucleases, the absence of which is consistent with other LGFs. Instead, the nucleic acid metabolism cassette encoded upstream of the putative NS2/3 could be a plausible proofreading candidate. One component of this cassette, deoxyribodipyrimidine photolyase is involved with nucleotide repair caused by UV damage (42). However, other components may serve different purposes. Adenylate kinases regulate adenosine phosphate concentrations (43), and, when present in dsDNA viruses, are thought to counterbalance the differential AT concentrations between the virus and its host along with NDPk (39). The genome of our divergent flavi-like virus exhibited a substantial AT bias (64%), and the nucleic acid metabolism cassette may in part function to sustain this. The only *Haliclona* genome available on NCBI (CAXHKC01) comprises ~58% AT. The lack of additional proofreading candidates reflected our inability to annotate or assess the role of large portions of the genome in silico because they did not share sequence or structural homology with known proteins. Isolation and culturing of this virus is needed to ascertain the function of these proteins. In vitro studies are also needed to estimate the intrinsic mutation rate of this virus so that it may be compared to those of canonical, ~10 kb vertebrate *Flaviviridae* (e.g., hepatitis C virus, dengue virus).

The genomic architecture of this virus may also be consequential in understanding the long-term evolutionary history of the *Flaviviridae.* In contrast to nidoviruses, which are thought to have undergone genomic expansion after the acquisition of a proofreading mechanism (19, 22, 44), members of the *Pestivirus*-LGF clade may have undergone genome reduction. The phylogenetic placement of NS5 sequences near the base of the *Pestivirus*-LGF clade was moderately well supported (ufboot = 92), and this position was consistent across multiple combinations of sequence alignment and alignment trimming methods. This tree topology would be consistent with the phylogenetic position of its putative host, a sponge, which was among the first animals to evolve (34). Thus, one interpretation of the size of the Maximus pesti-like virus genome is that it is a relic of an ancestral state of the pestiviruses, and the canonical (i.e., 10 kb) *Pestivirus* genome resulted from a series of genome reduction events, perhaps concordant with their emergence in vertebrates where longer genomes would mean more potential immune targets for organisms that had evolved adaptive immunity (45). If large sections of the *Pestivirus* genome were lost over time, it follows that the proteins encoded in these sections were selected against. This could explain why we were unable to find similar protein domains in other RNA viruses. It also raises the interesting possibility that some RNA viruses previously encoded a proofreading mechanism and then lost it, if one is found in the divergent flavi-like virus described here. Such a pattern would again be opposite to what has been inferred from the evolutionary history of the *Nidovirales.* Robust sampling of basal marine invertebrates for the purposes of virus discovery is required to address this hypothesis more thoroughly.

Another explanation for the aberrant size of this virus is that it belongs to a select group of specialized viruses that are successful “genetic pirates.” The acquisition of the nucleic acid metabolism cassette and the other functional domains could have been the result of relatively recent genetic piracy wherein an ancestral virus captured genetic material from a cellular organism. The divergent flavi-like virus appears to have acquired these domains in at least two separate events because its putative elongation factor does not share detectable sequence similarity with known cellular homologues, whereas its NDPk domain does. This suggests that the ancestors of this virus may have been adept at stealing domains from cellular organisms, a characteristic that may not be representative of most ancestral *Flaviviridae*. However, no conclusions can be drawn on the origin of the large sections of the genome that could not be aligned to any known member of the *Flaviviridae,* were not present in any RNA virus searchable in publicly available databases, and did not share structural homology with known cellular proteins. It may be that these sections were derived from organisms that are underrepresented in publicly available databases. Alternatively, they may have been captured long before the identifiable domains such that they no longer share detectable structural similarity with their cellular homologs. An assessment of this “genetic piracy” hypothesis will require the discovery of additional LGFs of comparable size and a more rigorous exploration of the LGF “foldome.”

Regardless of when it occurred, the apparent acquisition of domains sharing sequence and structural similarity to those found in bacteria is puzzling. Bacterial NADAR and Nudix domains have been found in RNA viruses, including among the *Nidovirales*, but these are thought to have been acquired through horizontal gene transfer from a eukaryote (18, 46). Although they did not have robust statistical support, the topologies of our NDPk phylogenetic trees are inconsistent with this sequence of events because in three of four topologies, the flavi-associated NDPk was most closely related to bacteria-associated NDPk domains (*SI Appendix*, Fig. S7). One explanation is that ancestral viruses infected bacteria-eating protists. Because bacteria have evolved defenses that allow them to use their would-be predators as safe ecological niches (47), coinfection with an ancestral flavi-like virus could have afforded the virus an opportunity to steal bacterial domains. Alternatively, sponges harbor many endosymbionts, including bacteria (48), which may have facilitated bacteria-virus gene transfer. It may also be that the flavi-associated NDPk shares closer sequence similarity to an unsampled eukaryote, and its position in the phylogenetic tree will change as new genetic data become available.

Filling sampling gaps in the RNA virosphere may shift paradigms in virus evolution if more exceptions to conventional wisdom are documented. Here, we present one such example by showing that flavi-like viruses can support genome sizes comparable to those observed in the *Nidovirales*. The absence of a detectable exonuclease domain demonstrates that other solutions are available to RNA viruses for overcoming theoretical error thresholds. Future studies may find that the *Nidovirales* and the *Flaviviridae* are not unique in their possession of large genomes. The continued exploration of RNA virus diversity is therefore key to revealing the patterns that shaped the long-term macroevolution of RNA viruses.

## Materials and Methods

### Sample Collection.

All invertebrate samples analyzed here (n = 72) were collected on October 20, 2022, in Chowder Bay, Sydney, Australia. Samples were collected by divers wearing latex gloves and using forceps cleaned with 96% ethanol between sampling events. All samples were placed in RNA/DNA-free cryogenic tubes, which were snap-frozen and stored immediately in liquid Nitrogen. Samples were then transferred to a −80 °C freezer.

### Sample Preparation and Metatranscriptome Sequencing.

Sample tissue was processed individually by flash freezing with liquid nitrogen and homogenizing with a mortar and pestle. Total RNA was extracted using the QIAGEN RNeasy Plus Mini kit. The RNA of some samples was pooled for downstream processing. The libraries in which the divergent flavi-like virus was found comprised single samples. Sequencing libraries were prepared using the Ribo-Zero Plus library preparation kit and sequenced on the Illumina NovaSeq 6000 platform. In total, 44 libraries were sequenced, including a “blank” negative control comprising a sterile water plus reagent mix.

### Virus Identification.

Raw reads were processed by removing sequencing adapters with Cutadapt v1.8.3 (49) and the parameters: -u 5 -U 5 -q 25 -m 25. The quality of trimming was assessed using Fastqc v0.11.8 (50). Reads mapping to rRNA were removed using SortMeRNA v4.3.3 (51) and the SILVA rRNA database (as of Dec. 2023). Contigs were assembled with MEGAHIT v1.2.9 (23) setting the minimum contig length to 200 nt). All contigs were screened against the RdRp-scan database (52) and a custom RNA virus databases using DIAMOND BLASTx v2.0.9 (53) with the setting ultrasensitive and an e-value cutoff of 1e-5. The custom database included divergent viruses identified in ongoing research projects. Virus candidates (i.e., viruses with a detectable sequence similarity to viruses in one or both of the databases) that were at least 1 kb in length were collated for verification. To verify that putative virus sequences were not misassigned host genes, all candidates were screened against the NCBI nonredundant protein database (as of Sept. 2023) using DIAMOND BLAST with an e-value cutoff of 1e-5 and the setting very-sensitive. Virus candidates sharing sequence similarity to host genes were excluded from further analysis. No virus candidates were detected in the negative control.

The sequence of the divergent flavi-like virus was translated using Expasy (https://web.expasy.org/translate/) to verify the presence of a complete and uninterrupted open reading frame. UTRs were manually annotated in Geneious Prime v2023.2.1. To further verify that this sequence did not contain known host gene sequences, the polyprotein was screened for sequence and structure homologies using BLASTp and Phyre2 (54). The assembly of the contig was compared against assemblies generated using SPAdes v3.15.5 (24) and Trinity v2.8.6 (25).

The abundance of the remaining reads (i.e., non-rRNA reads) was estimated using RSEM v.1.3.0 (55) implemented in Trinity v.2.5.1 (25). Raw reads were aligned with bowtie2 v2.3.31 (56). The abundance of the contig encoding the flavi-like virus described in this study was calculated by dividing the expected count for that contig by the sum of the expected count for all rRNA reads.

### Genome Sequencing Coverage.

Sequencing coverage was assessed using BBMap v37.98 (26) with a mapping threshold of 95%. Calculation of the Q30 score and mapping visualization was performed by Geneious Prime (www.geneious.com).

### Host Associations.

To characterize the host composition of the library, non-rRNA reads and contigs were first screened using KMA v1.3.9a (57) and CCMetagen v1.1.3 (58). Reads were mapped to the prebuilt KMA database. Next, rRNA reads identified with SortMeRNA v4.3.3 (51) were assembled using MEGAHIT v1.2.9 (23) and screened against the NCBI Blast nt database (as of December 2023).

### Strand-Specific Molecular Analysis.

#### cDNA Synthesis by Reverse Transcription.

The first (+) and (−) strands were generated by reverse transcription using the SuperScript™ IV First-Strand Synthesis System (ThermoFisher). Reactions were performed using 100 ng of RNA as the template, 1 μL of the appropriate strand-specific primer flanked with a nonviral sequence tag (2 μM, Table 1), and 1 μL dNTPs (10 mM). The mixture was heated at 65 °C for 5 min and incubated on ice for 5 min. In a separate tube, 4 μL of SSIV Buffer (5X) was mixed with 1 μL of DTT (100 mM), 1 μL of Ribonuclease Inhibitor and 200 units of Superscript IV Reverse Transcriptase (Invitrogen). The RNA-primer mix and the RT reaction mix were mixed together and incubated at 55 °C for 30 min and subsequently inactivated by heating at 80 °C for 10 min. cDNAs were then used for the end-point and qPCR.

**Table 1.** Primers used for the standard RNA transcripts, RT, and PCR for the strand-specific assay

RT reaction	Name	Sequence 5′–3′	Position (nt)
(+) strand	Tag Pos RT	CGGGAAGGCGACTGGAGATCATATGAGCATTCAGAGTACC	4790
(−) strand	Tag Neg RT	CCGTCATGGTGGCGAATAAGACTGGTGTCAATTAGAAGAATC	4700
**PCR and qPCR**			
(+) strand	PCR Pos F	GACTGGTGTCAATTAGAAGAATC	4700
	PCR Pos R	CGGGAAGGCGACTGGAG	Tag
(−) strand	PCR Neg F	CCGTCATGGTGGCGAATAA	Tag
	PCR Neg R	ATCATATGAGCATTCAGAGTACC	4790
**Standard RNA**			
(+) strand	T7 Pos F	**GCGTAATACGACTCACTATAG**GCTTCGATTCAAGATTACGTTTA	4586
	T7 Pos R	GATCCAAAGTGTCCATGTATGA	5564
(−) strand	T7 Neg F	GCTTCGATTCAAGATTACGTTTA	4586
	T7 Neg R	**GCGTAATACGACTCACTATAG**TAGAGATCCAAAGTGTCCATGTATGA	5564

Underlined nucleotides represent the unique nonviral tag sequence for the strand-specific RT reaction. Sequences in bold indicate the T7 promoter for the standard RNA generation. (+): positive; (−): negative; nt : nucleotides.

#### End-Point PCR Amplification.

cDNAs produce by RT were used as templates for amplification in PCR carried out in a final volume of 50 μL that included 25 μL of Platinum™ SuperFi™PCR Master Mix (2X, ThermoFisher), 2.5 μL of each respective forward and reverse primers (10 μM), 10 μL of cDNA, and 10 μL of nuclease-free water. PCRs were performed on a SimpliAmp™ (ThermoFisher) thermocycler with the following conditions: 98 °C for 30 s followed by 45 cycles of 98 °C for 10 s, for 61 °C for 10 s, 72 °C for 10 s, and 72 °C for 5 min. The PCR products were analyzed on SYBR Safe (ThermoFisher) stained agarose gels.

#### Strand-Specific RNA Transcripts.

Synthetic positive and negative RNA transcripts standards were synthetized by in vitro transcription using T7 RNA polymerase. First, the positive and negative cDNA products were produced by RT-PCR from the extracted RNA, using their respective primer pairs containing a T7 promoter at the 5′ end (Table 1). Second, the in vitro transcription was performed using the HiScribe™ T7 High Yield RNA Synthesis Kit (NEB). Briefly, 10 μL of the reaction buffer (10X) was mixed with 2 μL of ATP, UTP, GTP, CTP (100 mM each), 1 μL of DTT (0.1 M), 2 μL of T7 RNA polymerase mix, 2 μL of T7-DNA products (1 μg) and 5 μL of nuclease-free water. The reaction was incubated at 37 °C for 2 h and RNA transcripts were then purified using the RNeasy MinElute Cleanup Kit (Qiagen). Concentrations of purified RNA were determined using a NanoDrop spectrophotometer (ThermoFisher).

#### Strand-Specific qPCR Assay.

The QuantiNova SYBR Green PCR Kit (Qiagen) was used for the strand-specific quantification by qPCR. The mixture contained 10 μL of SYBR Green PCR Master Mix (2×), 1.25 μL of forward primer and reverse primers (0.7 μM), 6 μL of cDNA, and 2.5 μL of nuclease-free water. Assays were performed using the LightCycler® 480 Instrument II (Roche) with the following conditions: 95 °C for 2 min, followed by 40 cycles of 95 °C for 5 s, 60 °C for 10 s, 72 °C for 30 s, and data collection occurred during the 60 °C step. Synthetic RNA representing each strand, serially diluted from 10^11^ to 10^2^ was used to calculate the amount of viral RNA copies.

### Annotation of Functional Domains.

Preliminary annotation of the divergent flavi-like virus was performed using InterProScan v2.1 against the CDD, SuperFamily, and NCBIfam databases as implemented in Geneious Prime v2023.2.1. For manual annotation of protein boundaries, transmembrane (TM) domains were predicted using DeepTMHMM v1.0.24 (59). These TM domains were then analyzed to determine whether they were signal peptidases by querying a 40 amino acid (aa) region encompassing each TM domain using a 15aa sliding window with SignalP v6.0 (60). The potential N-linked glycosylation residues on the polyprotein were identified using NetNGlyc v1.0 (61), with likely N-linked glycan residues considered above the threshold of 0.5. For functional protein domain prediction, the polyprotein sequence was queried against the Pfam v34.0 (62) and CDD v3.20 (63) databases utilizing the NCBI CD-search program (64) with an e-value threshold of 0.1. To examine the presence of a protease, we aligned with NS3Pro of Classical swine fever virus (CSFV), Pangolin pestivirus, and the divergent flavi-like virus with MAFFT v7.511 (65) L-INS-I method. CSFV was chosen because it is a pestivirus and the catalytic triad in its protease is well documented. According to BLASTp, the highest sequence similarity to the putative NS3 region of the divergent flavi-like virus was found in Bovine viral diarrhea virus 3 (AFL65618.1). The putative envelope protein was identified using HHpred (66), which predicted its presence with 60.1% probability when the first 6,000 amino acids of the polyprotein were tested against the PDB_mmCIF70_24_Oct database.

### Protein Structure Prediction and Homology Search.

We exploited machine-learning approaches that allow high-confidence ab initio protein structure prediction from sequence data alone. The polyprotein sequence of the divergent flavi-like virus (12,694 amino acids) was split into 300 residue blocks each overlapping by 100 residues (resulting in a total of 125 sequence blocks; *SI Appendix*, Fig. S6*A*). The structure of each sequence block was predicted using both ColabFold-AlphaFold2 (29, 30) and ESMFold (31) (*SI Appendix*, Fig. S6 *B* and *C*). While the majority of the polyprotein did not yield confident predictions, peaks in confidence corresponding to well-folded domains were produced by both methods. ESMFold performed somewhat better than ColabFold (*SI Appendix*, Fig. S6 *D*–*F*), likely due to the latter’s requirement for multiple sequence alignments, which cannot be readily assembled for highly divergent sequences, such as those found in this virus. Structure-guided homology searches were performed using the Foldseek server (32) and we focused on hits against experimentally solved structures from the protein database (PDB). Additionally, we used a local installation of Foldseek to query a protein structure prediction database covering the entire *Flaviviridae*, as described by Mifsud et al (27). We evaluated the distribution of the identity of Foldseeks hits to ensure that the reported top hit for each inferred structure reflected the majority of hits obtained through this screen, and we assessed concordance between Foldseek hits returned when either ESMFold or ColabFold inferred structures were used as input (*SI Appendix*, Fig. S6*D*). Tables of Foldseek hits for each structure block shown in the main figures are provided in Datasets S2 and S3.

### Phylogenetic Analysis.

For the family-level virus analysis, all *Flaviviridae* (taxid 11050 and taxid 38144) available on the NCBI Virus were downloaded on Dec. 15, 2022. These data were supplemented with sequences from Mifsud et al. (67), Mifsud et al. (27), and the NCBI nucleotide database (searched using the phrase “flavi[All Fields] OR pesti[All Fields] OR hepaci[All Fields] OR pegi[All Fields] AND viruses[filter]” on Dec. 15, 2022). We screened the BLASTx results for the divergent flavi-like virus for sequences published in 2023 and added the two that met these criteria (UHR49738.1 and WPA70758.1) to the dataset. We also screened the NCBI Virus database for pesti- and pesti-like viruses and large-genome flavi-like viruses published in 2023 that were longer than 20 kb, and consequently added Macrosiphum euphorbiae virus 1 isolate K01 (YP_009175071, length = 22.8 kb) to the dataset. Redundant nucleotide sequences were removed using CD-HIT (68) at a cutoff of 95%. Remaining sequences were translated using the Geneious Prime Find ORFs tool (v.2022.0) (www.geneious.com) and trimmed to the NS2/3 and NS5 regions, individually. Putative NS5 sequences that lacked the conserved GDD palm motif were excluded. Incomplete genomes were removed manually. The corresponding segments of the two closest protein BLASTp hits to the divergent flavi-like virus (Bovine viral diarrhea virus 3 [accession ANW09737] and *Pestivirus brazilense* [accession WEC89329]) were added to each dataset. Members of the *Potyviridae* were used as the outgroup for both trees. The final NS2/3 and NS5 datasets contained 427 and 464 sequences, respectively. All datasets were aligned with MAFFT v7.490 (65) using default parameters and the BLOSUM62 scoring matrix (NS2/3 length = 2,279aa; NS5 length = 2,537aa). Ambiguously aligned regions were removed with a conservation score of 0.15 using trimAl v1.4.1 (69).

For the genus-level analysis, pestivirus and pesti-like virus sequences were realigned using members of the genus *Orthoflavivirus* as the outgroup. Both MAFFT v7.490 (65) and MUSCLE v5.1 (70) alignment algorithms were used. Ambiguously aligned regions were removed at a range of thresholds (Table 2) using trimAl v1.4.1 (69).

**Table 2.** Combinations of sequence alignment and trimming methods used in phylogenetic analysis of the *Pestivirus*-LGF clade

Protein	Alignment method	trimAl parameters	Alignment length (aa)	Substitution model selected
NS5	MAFFT	N/A	3,317	LG+F+R10
NS5	MUSCLE	N/A	6,085	LG+R10
NS5	MAFFT	-gappyout	621	LG+F+R10
NS5	MUSCLE	-gappyout	453	LG+F+R8
NS5	MAFFT	-gt 0.15 -cons 0.15	1,146	LG+F+R10
NS5	MUSCLE	-gt 0.15 -cons 0.15	1,178	LG+F+R10
NS2/3	MAFFT	N/A	3,321	LG+F+R6
NS2/3	MUSCLE	N/A	8,477	LG+F+R10
NS2/3	MAFFT	-gappyout	971	LG+F+R10
NS2/3	MUSCLE	-gappyout	1,016	LG+F+R10
NS2/3	MAFFT	-gt 0.15 -cons 0.15	1,525	LG+F+R10
NS2/3	MUSCLE	-gt 0.15 -cons 0.15	1,226	LG+F+R10

All phylogenetic trees were inferred using the maximum likelihood method available in IQ-TREE v1.6.12 (71) with 1,000 ultrafast bootstraps. ModelFinder Plus was enabled for inference of the family-level phylogenies (NS3: LG+F+R8, NS5: LG+F+R10), while substitution model selection was restricted to LG models for the *Pestivirus*-LFG clade because of the selection for the family-level analysis.

To infer the NPDk phylogenetic tree, the top 500 BLASTp results to the flavi-associated NDPk domain were downloaded. Redundant sequences were removed with CD-HIT (68) v4.8.1 at a threshold of 90%, leaving 139 sequences for subsequent analysis. Sequences were aligned with MAFFT v7.490 (65) and MUSCLE v5.1 (70), and the ends were manually trimmed to preserve the most conserved region (length = 269 amino acids, gaps inclusive). Ambiguously aligned regions were removed using trimAl v1.4.1 (69) with the setting “gappyout.” This resulted in four alignments: i) MAFFT without ambiguities removed, ii) MAFFT with ambiguities removed, iii) MUSCLE without ambiguities removed, and iv) MUSCLE with ambiguities removed. Maximum likelihood trees were inferred using IQ-TREE v1.6.12 (71) with 1,000 SH-aLRT bootstraps and 1,000 ultrafast bootstraps. The ModelFinder was unrestricted. Selected models are shown in *SI Appendix*, Fig. S7.

## Supplementary Material

Appendix 01 (PDF)

Dataset S01 (XLSX)

Dataset S02 (XLSX)

Dataset S03 (XLSX)

## Data Availability

Alignments and trees referred to in this manuscript as well as the nucleotide and amino acid sequences of the flavi-like virus are available on GitHub (https://github.com/mary-petrone/large_flavi) (72). All other data are included in the manuscript and/or supporting information.
