# Supplementary material for: A ~40-kb flavi-like virus does not encode a known error-correcting mechanism
Source: Proc Natl Acad Sci U S A. 2024 Jul 17;121(30):e2403805121. doi: 10.1073/pnas.2403805121 (PMC11287256; doi:10.1073/pnas.2403805121)
Supplement: Supplementary file 1 — Appendix 01 (PDF) [file pnas.2403805121.sapp.pdf]

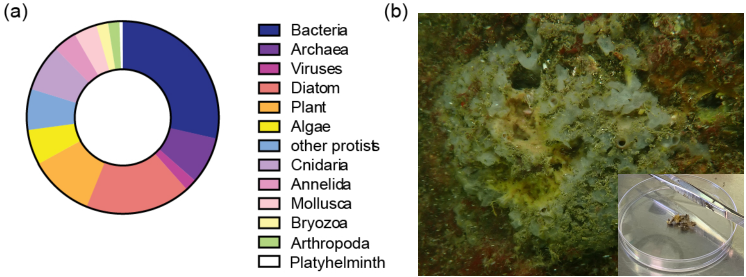

Supplementary Figure 1. Complex host composition of sequencing library. (a) Library composition inferred using KMA and ccmetagen. (b) Photograph of piling ecosystem at the time of sample collection and of sample at time of processing (*inset*).

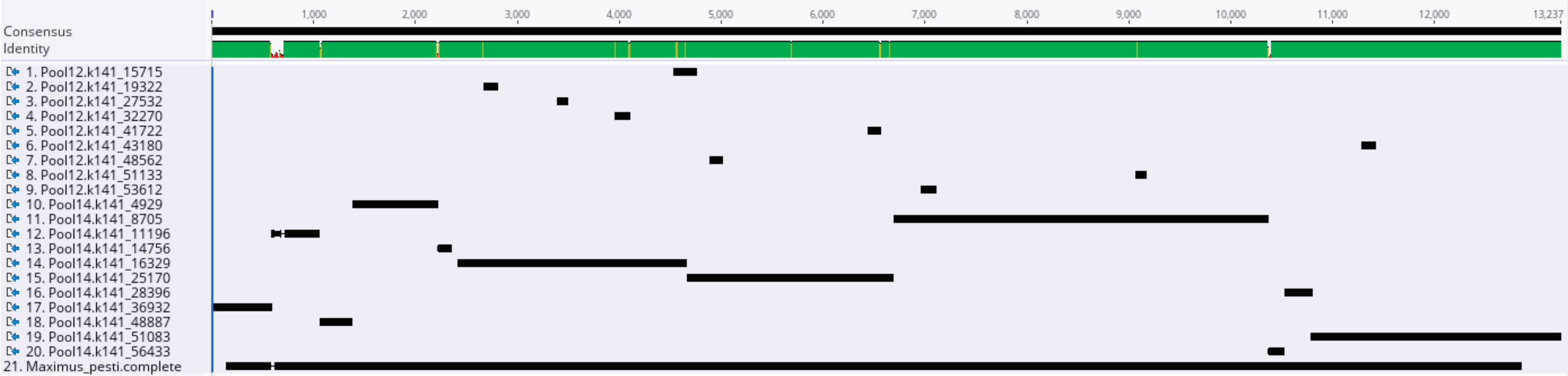

Supplementary Figure 2. Alignment of Maximus pesti-like virus fragments identified in three sequencing libraries. Sequences were aligned with CLUSTAL Omega[26] . The alignment was visualised in Geneious Prime. The additional pools are denoted by number (Pool 12 and Pool 14).

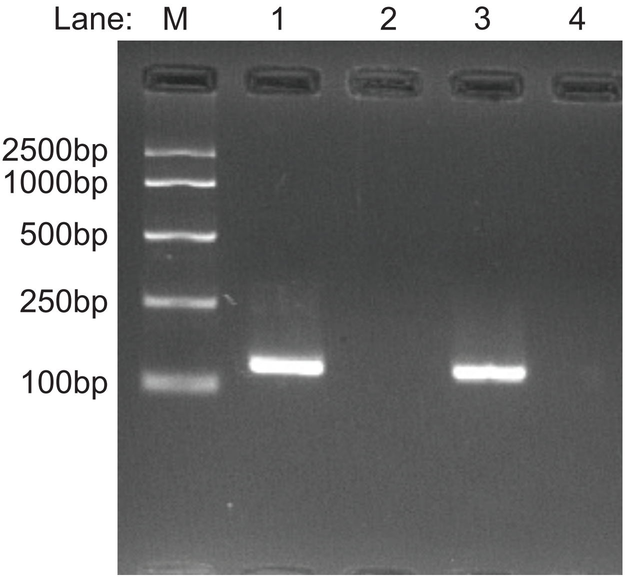

Supplementary Figure 3: Gel electrophorsesis of tag-based end-point PCR for strand-specific detection.

Lanes 1 and 3: Tag-PCR amplification of positive (lane 1) and negative (lane 3) strands of “Maximus pesti-like virus”.
Lanes 2 and 4: PCR amplification of the positive (lane 2) and negative (lane 4) strands without their respective tags.
M: Molecular marker

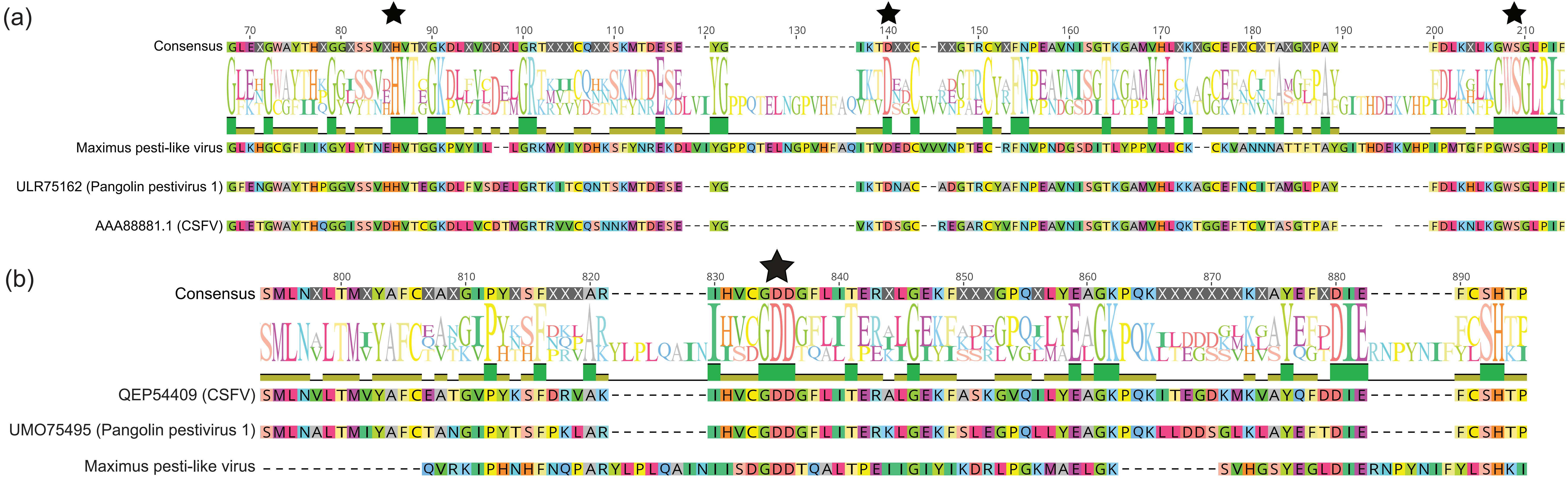

Supplementary Figure 4. Amino acid alignments of conserved domains in NS2/3 and NS5. (a) Alignment of Ser (nucleophile), His (base), Asp (acid residue) catalytic triad. (b) Conservation of the GDD motif in the NS5 protein. Key amino acids are denoted with a star icon. Sequences were aligned with MAFFT. Images were rendered in Geneious Prime and formatted in Adobe Illustrator.

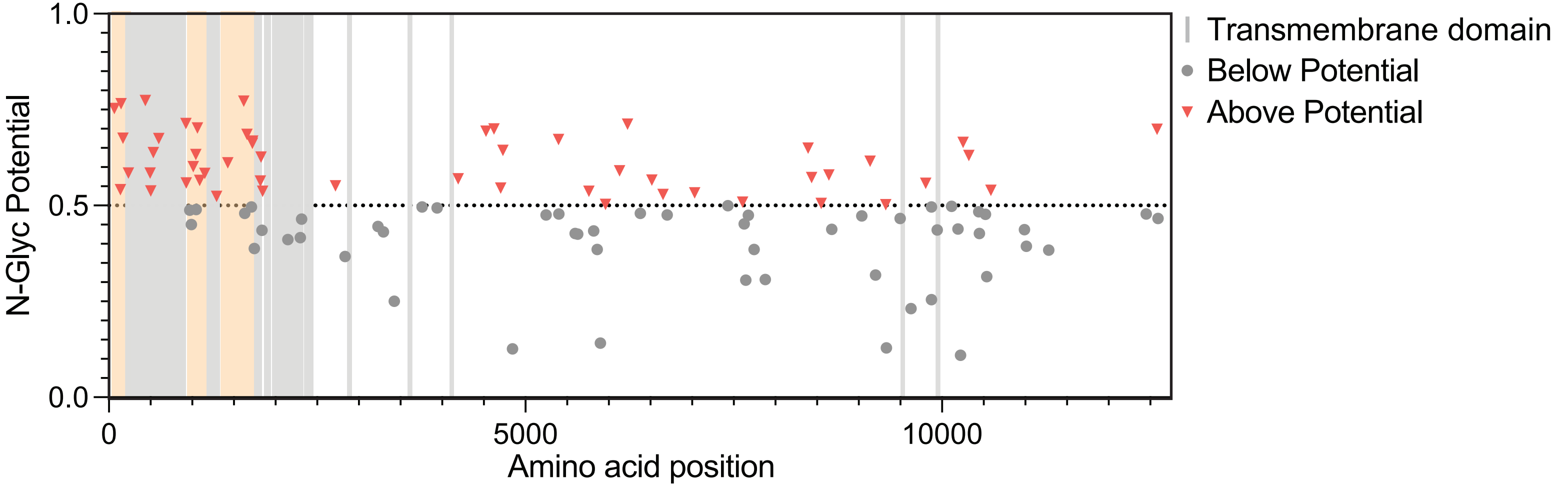

Supplementary Figure 5. Predicted N-linked glycosylation sites. Potential N-linked glycosylation residues on the polyprotein were identified using NetNGlyc v1.0[61], with likely N-linked glycan residues considered above the threshold of 0.5. Data were visualised in GraphPad Prism v10.1.0.

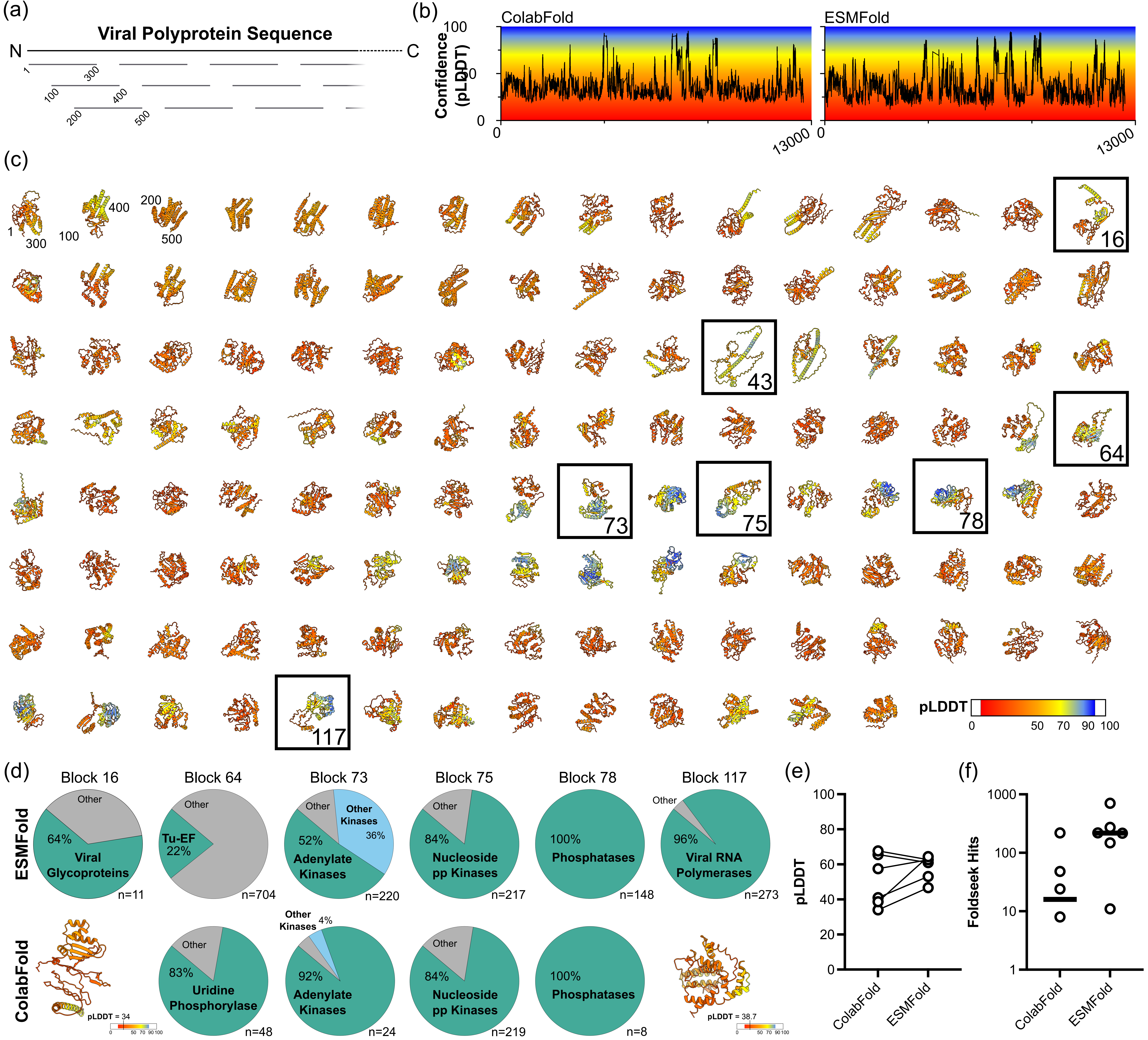

Supplementary Figure 6. Protein structure prediction and homology search. (a) Schematic illustrating the overlapping sequence blocks used for structure prediction, residue numberings are shown for the first three blocks. (b) Average prediction confidence plots (predicted Local Distance Difference Test, pLDDT) along the length of the viral polyprotein for structures generated using ColabFold and ESMFold. (c) ESMFold structure predictions for all 125 sequence blocks color-coded by prediction confidence (pLDDT) as shown in the key and in (b). Blocks that appear in main text figures are outlined and numbered. The N- and C-termini are numbered by residue for the first three blocks, as in (a). (d) Pie charts displaying the frequency of the most prevalent Foldseek hit for each of the sequence blocks, predicted either by ESMFold or ColabFold (except block 43, which exhibits no structural homology). The hits were filtered for e-values below 0.01, and the resultant number of hits (n) is provided for each chart. ColabFold yielded very low confidence predictions for blocks 16 and 117 and, consequently, no homology was detected; structures are shown in place of the respective pie charts. (e) Average pLDDT confidence for each of the sequence blocks predicted by ColabFold and ESMFold. (f) Number of Foldseek hits for ColabFold and ESMFold structures, circles represent individual sequence blocks, horizontal lines the mean.

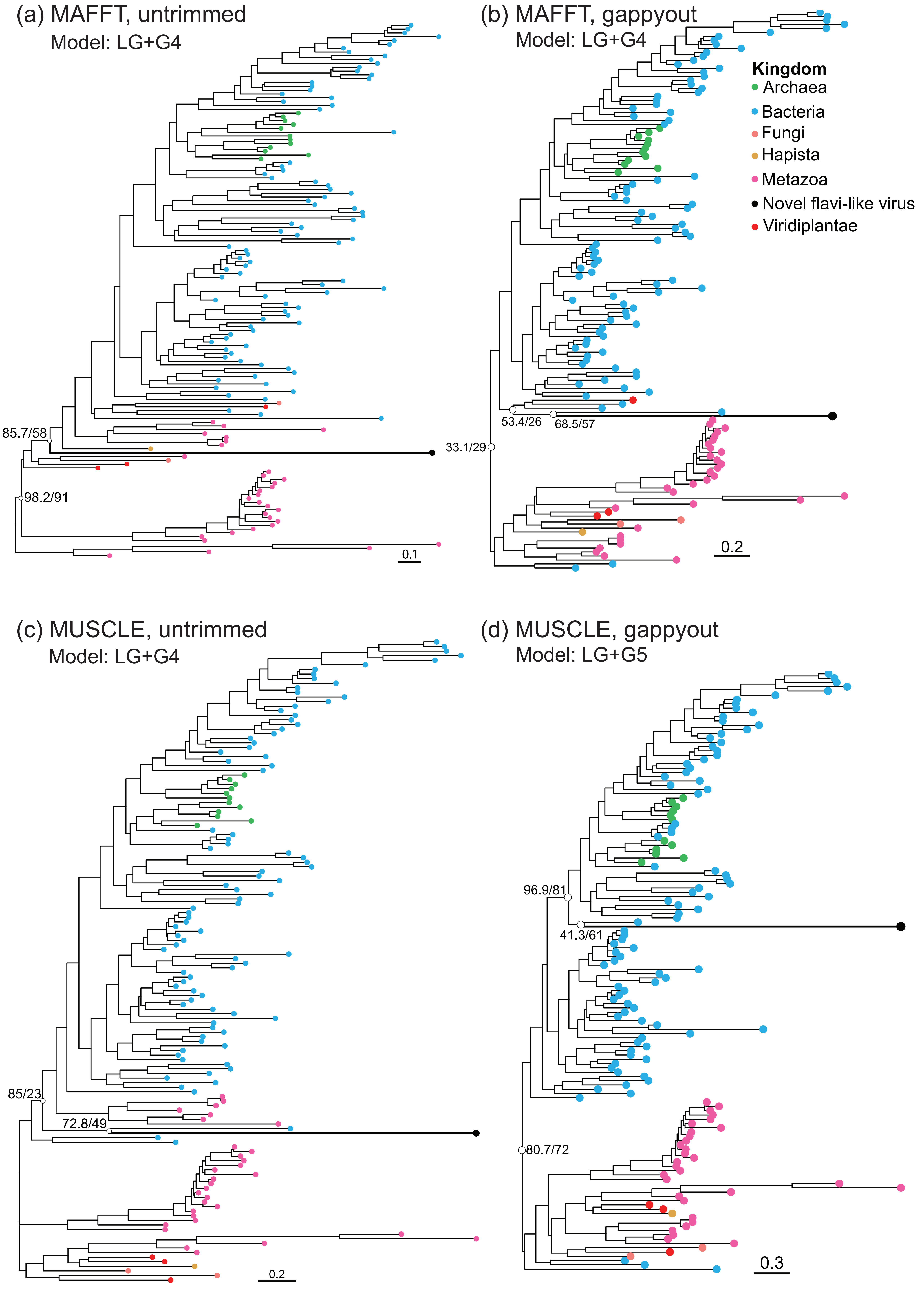

Supplementary Figure 7: Combinations of aligning and trimming methods of NDPk phylogenetic inference. All trees are midpoint rooted. MAFFT v7.490 and MUSCLE v5.1 were used to align the sequences. Ambiguities were removed with trimAl v1.4.1 with the 'gappyout' flag where indicated.Tree branches are scaled according to amino acid substitutions. SH-aLRT and ultrafast bootstrap support are shown at select nodes (SH-aLRT/UFboot).

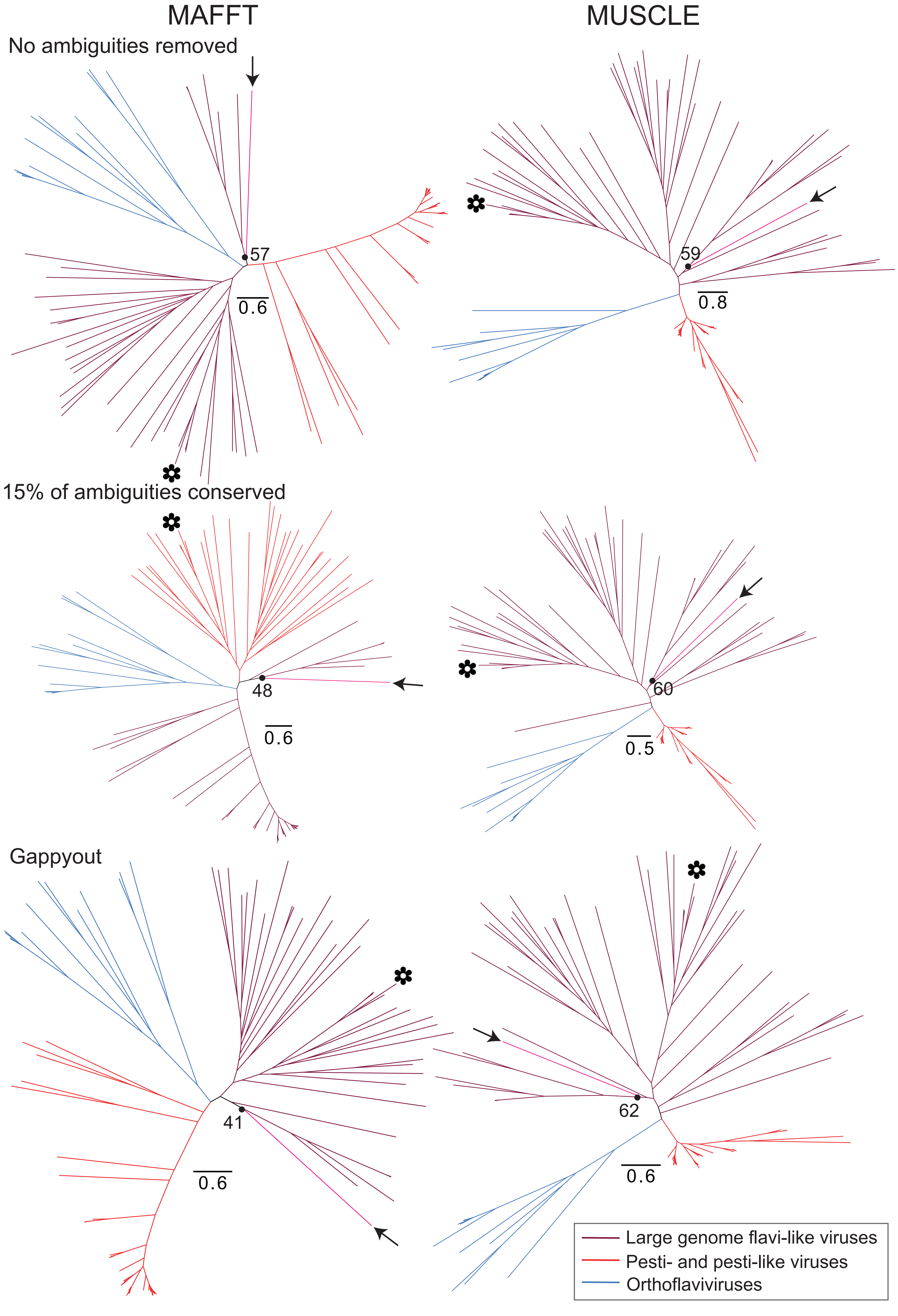

Supplementary Figure 8. Unrooted, maximum likelihood phylogenetic trees of selected Flaviviridae NS2/3 inferred with IQ-TREE from six combinations of alignment and trimming methods. Arrows indicate placement of the novel flavi-like virus. Flower icons indicate the placement of plant-associated viruses. Node values indicate ufboot support of this placement.

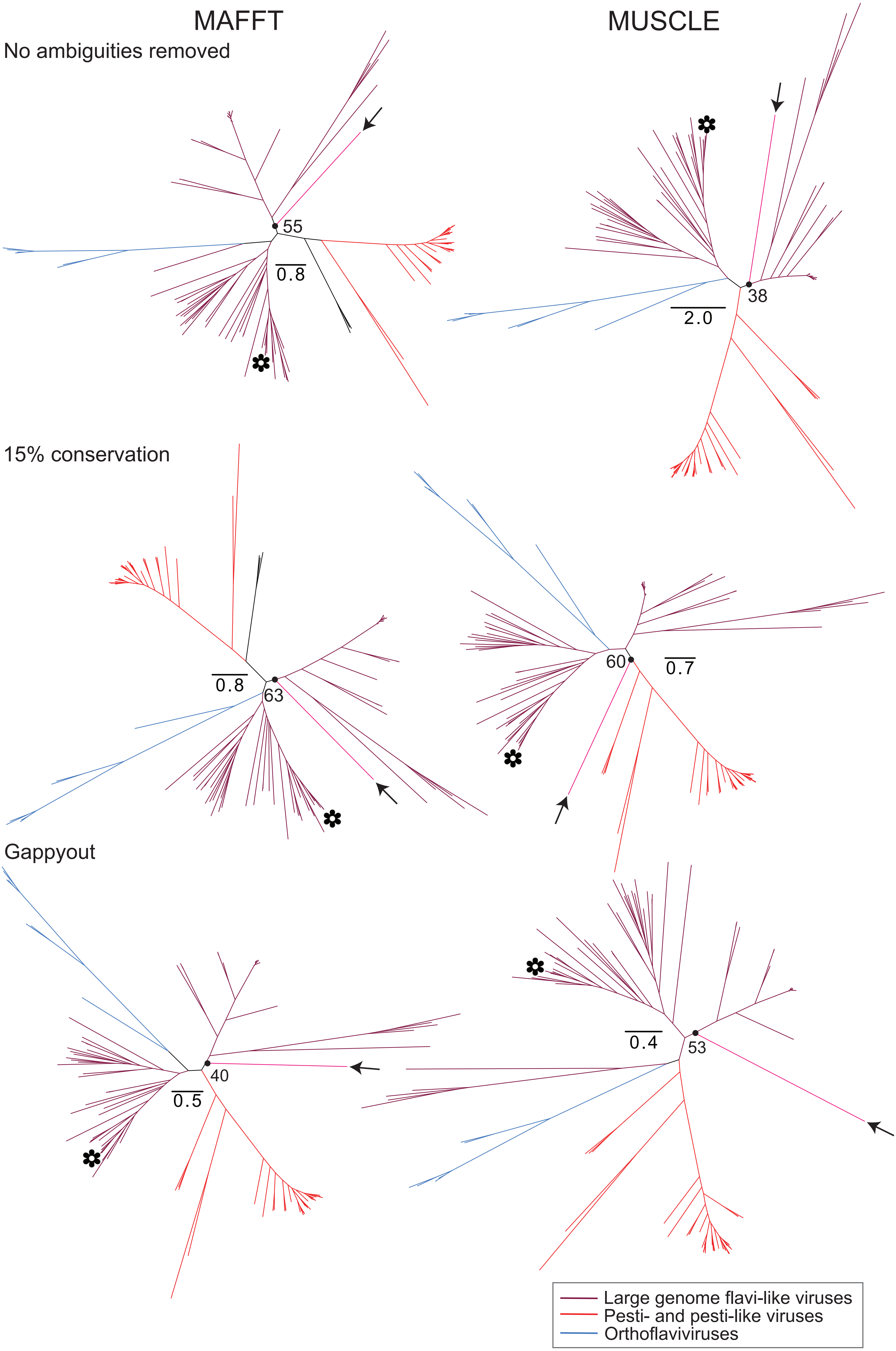

Supplementary Figure 9. Unrooted, maximum likelihood phylogenetic trees of selected *Flaviviridae* NS5 inferred with IQ-TREE from six combinations of alignment and trimming methods. Arrows indicate placement of the novel flavi-like virus. Flower icons indicate the placement of plant-associated viruses. Node values indicate ufboot support of select nodes.
